# The role of intestinal microbiota and its metabolites in intestinal and extraintestinal organ injury induced by intestinal ischemia reperfusion injury

**DOI:** 10.7150/ijbs.71491

**Published:** 2022-06-13

**Authors:** Fan Deng, Ze-Bin Lin, Qi-Shun Sun, Yue Min, Yue Zhang, Yu Chen, Wen-Ting Chen, Jing-Juan Hu, Ke-Xuan Liu

**Affiliations:** Department of Anesthesiology, Nanfang Hospital, Southern Medical University, Guangzhou, Guangdong, China.

**Keywords:** Intestinal microbiota, Metabolites, Intestinal ischemia reperfusion, extraintestinal organ injury

## Abstract

Intestinal ischemia/reperfusion (I/R) is a common pathophysiological process in clinical severe patients, and the effect of intestinal I/R injury on the patient's systemic pathophysiological state is far greater than that of primary intestinal injury. In recent years, more and more evidence has shown that intestinal microbiota and its metabolites play an important role in the occurrence, development, diagnosis and treatment of intestinal I/R injury. Intestinal microbiota is regulated by host genes, immune response, diet, drugs and other factors. The metabolism and immune potential of intestinal microbiota determine its important significance in host health and diseases. Therefore, targeting the intestinal microbiota and its metabolites may be an effective therapy for the treatment of intestinal I/R injury and intestinal I/R-induced extraintestinal organ injury. This review focuses on the role of intestinal microbiota and its metabolites in intestinal I/R injury and intestinal I/R-induced extraintestinal organ injury, and summarizes the latest progress in regulating intestinal microbiota to treat intestinal I/R injury and intestinal I/R-induced extraintestinal organ injury.

## Introduction

Intestinal ischemia/reperfusion (I/R) injury is common in the perioperative period, especially in both surgical and trauma patients, and has high morbidity and mortality associated with it 1-3. Intestinal I/R plays an important role in the pathophysiological evolution of severe infection, trauma, shock, intestinal obstruction, mesenteric artery embolism, abdominal aortic aneurysm surgery, cardiopulmonary bypass surgery, liver transplantation, and small intestine transplantation 4, 5. It not only causes local intestinal injury, but can also disrupt intestinal mucosal barrier, which allows enteric bacterial endotoxin and various locally produced free radicals to penetrate the blood, translocate into the peripheral organs, and cause extraintestinal multiple organ dysfunction or even failure 6-8. Intestinal microbiota refers to the trillions of microorganisms that exist in the gastrointestinal tract, including bacteria, viruses, fungi, archaea and protozoa 9-14. They can interact with the host in a variety of ways and play a critical role in nutrient metabolism, xenobiotics and drug metabolism, maintenance of the intestinal barrier, and the structure and function of the gastrointestinal tract 15-19. In the past few decades, the research on intestinal microbiota and its metabolites and intestinal I/R injury and intestinal I/R-induced extraintestinal organ injury has increased rapidly. In this review, we focus on the role of intestinal microbiota and its metabolites in the occurrence, development, and prevention of intestinal I/R injury and intestinal I/R-induced extraintestinal organ injury, and provide guidance for the prediction, diagnosis and treatment of intestinal I/R injury in the future.

## Synopsis of intestinal I/R injury

Perioperative acute intestinal I/R injury is a common emergency and critical condition 2. It not only causes damage to the intestinal barrier, but can also lead to multiple organ injury outside the intestine, with a very high complication rate and mortality rate 20. Therefore, prevention and treatment of acute intestinal I/R injury during the perioperative period is of great significance to improve patient outcomes. As shown in Table 1, the factors that cause acute ischemic intestinal injury during the perioperative period can be divided into patients, anesthesia and surgery-related factors. Patient factors include advanced age, American Society of Anaesthesiologists (ASA) grade ≥ III, preoperative gastrointestinal disease or other diseases that lead to impaired gastrointestinal function (such as severe infection, acute severe pancreatitis, trauma, shock, anemia, myocardial infarction, aortic dissection, mesenteric artery embolism, etc.). Anesthetic factors include hypotension and intestinal hypoperfusion caused by anesthetics; contraction of small blood vessels in the gastrointestinal mucosa caused by some vasoconstrictor drugs induces intestinal ischemia; the sympathetic nervous system is excited under stress, intestinal mucosal blood vessels contract strongly and blood perfusion is reduced. Surgical factors include abdominal aortic aneurysm surgery, cardiopulmonary bypass (CPB), abdominal surgery and other intestinal operations that affect intestinal blood perfusion; CO_2_ pneumoperitoneum can cause stress response, and plasma catecholamine, cortisol, and antidiuretic hormone levels increase during laparoscopic surgery; at the same time, the increase in abdominal pressure affects the blood perfusion of internal organs. The intestine is the body's largest endotoxin reservoir and microbial reservoir. Once intestinal injury occurs, endotoxin and flora shift, secondary to endotoxemia and sepsis, resulting in multiple organs throughout the body (lung, brain, liver, etc.) dysfunction and even failure. However, the prediction of intestinal I/R injury still lacks effective biomarkers; diagnosis lacks uniform standards; treatment lacks effective drugs and measures. In recent years, the intestinal microbiota has been proved to play an important role in the occurrence, development, prediction, diagnosis and treatment of diseases. Therefore, summarizing the changes and potential roles of intestinal microbiota and its metabolites in intestinal I/R injury is of great significance for finding predictive and diagnostic biomarkers, new treatment methods and drugs for intestinal I/R injury.

## Overview of the intestinal microbiota

The human intestinal microbiota refers to the general name of the microorganisms inhabiting the human digestive tract, including bacteria, archaea, viruses, fungi and protists, etc., most of which are bacteria 21-25. There are direct or indirect interactions between them, and they form a complex interaction network with the host through direct contact, secreting proteins or metabolites, forming a dynamically balanced micro-ecosystem, which is closely related to human health and disease 26-29. Existing studies have shown that intestinal microbiota is closely related to the occurrence and development of diabetes, hypertension, cardiovascular disease, tumors, etc., because of its important role in human health, it is also called the human body's forgotten organs 9, 30-36. In addition, the intestinal microbiota is huge, and it contains about 100 times the number of genes in the human genome, so it is also known as the second genome of the human body 17, 37, 38. The main proportion of bacteria are *Firmicutes* and *Bacteroides*, which account for more than 90%. The relative abundance of *Proteobacteria* and *Eubacteria* is relatively low 39. The overall structure and function of the intestinal microbiota are stable for a period of time, but they are highly sensitive to changes in internal and external environments. Exogenous factors such as diet, exposure to bacterial infections or taking drugs can reduce the diversity of the intestinal microbiota; endogenous factors such as acute body fluid imbalance, chronic intestinal congestion or ischemia hypoxia, acid-base imbalance, gastrointestinal weakened exercise and nutritional deficiencies can potentially change the intestinal microbiota 40-44. Therefore, it is necessary and meaningful to summarize the changes of intestinal microbiota and its metabolites caused by intestinal I/R and the role of intestinal microbiota and its metabolites in intestinal I/R injury.

Studies have confirmed that intestinal microbiota and its metabolites play a variety of important roles and functions in our normal life activities, including nutrient absorption, growth and development, biological barrier, immune regulation, fat metabolism, anti-tumor, etc 45-47. The human intestinal microbiota is stimulated by large number of dietary nutrients to produce bioactive compounds such as bile acids, short-chain fatty acids (SCFA), ammonia, phenols, and endotoxins 18, 48-50. These microbial-derived metabolites are the communication medium between the microbe and the host, which is essential to maintain the normal physiological state of the host. As mentioned earlier, most of the human microbiota, especially the intestinal microbiota, cannot be isolated and cultured purely, which makes it difficult for traditional microbiological research methods to carry out research on the human microbiota. With the advent of metagenomics, important breakthroughs have been made in the study of intestinal microbiota. At present, the most direct and efficient method to detect the composition of the microbial community is to use amplicon sequences for target genes, such as 16S rRNA gene sequencing to identify the composition of bacterial communities, and Internally Transcribed Spacer (ITS) sequencing to identify the composition of fungal communities. However, amplicon sequencing cannot provide the genetic information carried by the flora. Currently, metagenomic sequencing is mainly used, that is, to sequence the genomes of various microorganisms including bacteria, fungi, viruses, etc., to identify the genetic information carried by the microbiota, and to analyze the functions of genes and possible metabolic pathways. In order to further examine the functions of these microbial groups, multi-omics research is more used. Metatranscriptome is used to detect the structure of functionally active microbial populations at the transcriptional level 51, 52; metaproteome is used to detect protein information translated by functionally active microbial populations 17, 53; metabolome is used to detect metabolite information produced by functionally active microbial populations 46, 54.

## Changes in intestinal microbiota and metabolites induced by intestinal I/R

**Figure 1:**
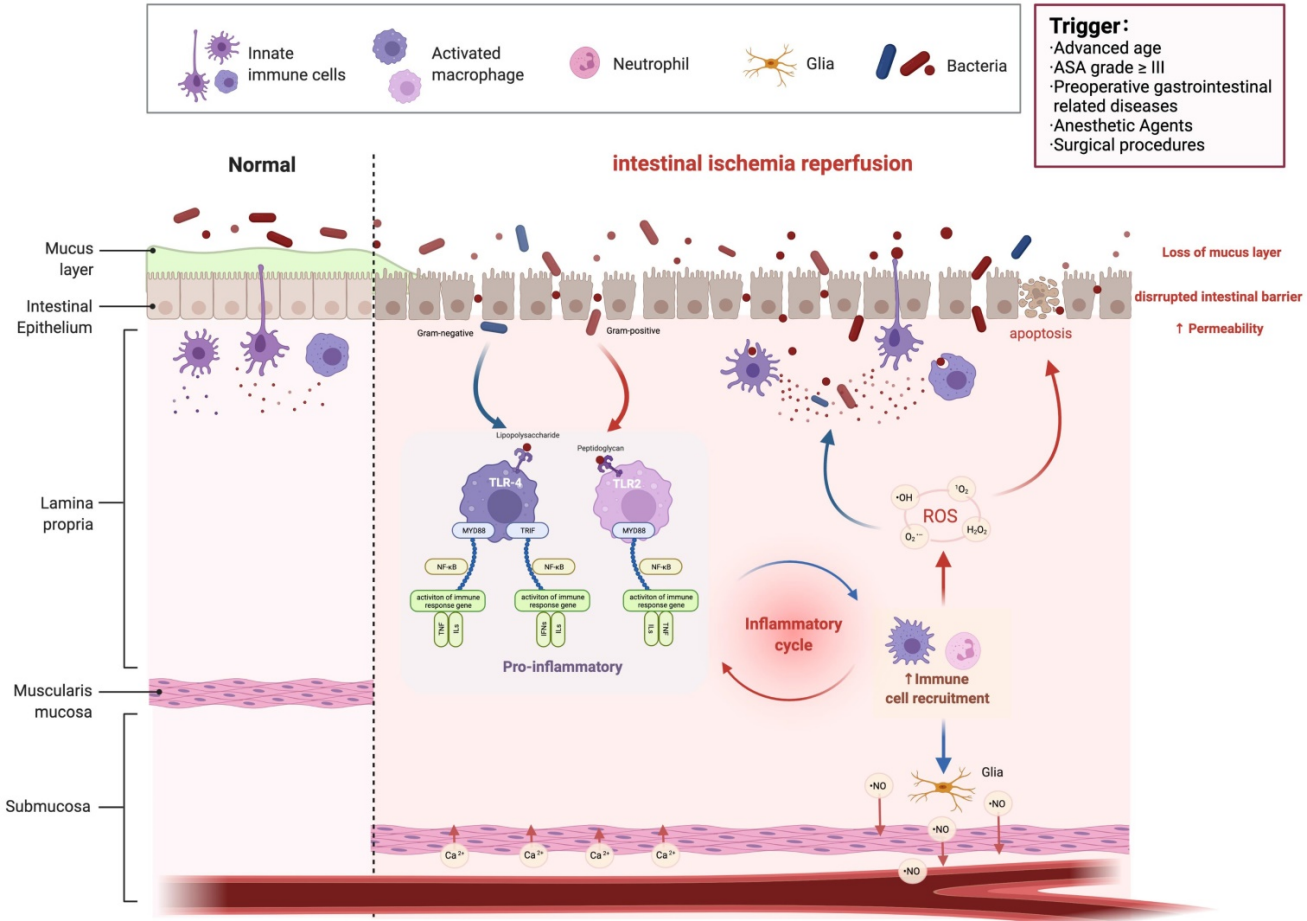
** Intestinal I/R injury.** I/R, ischemia reperfusion; ASA, American Society of Anesthesiologists; TLR, Toll-like receptors; MYD88, Myeloid differentiation factor 88; ROS, Reactive oxygen species; TNF, tumor necrosis factor.

**Figure 2:**
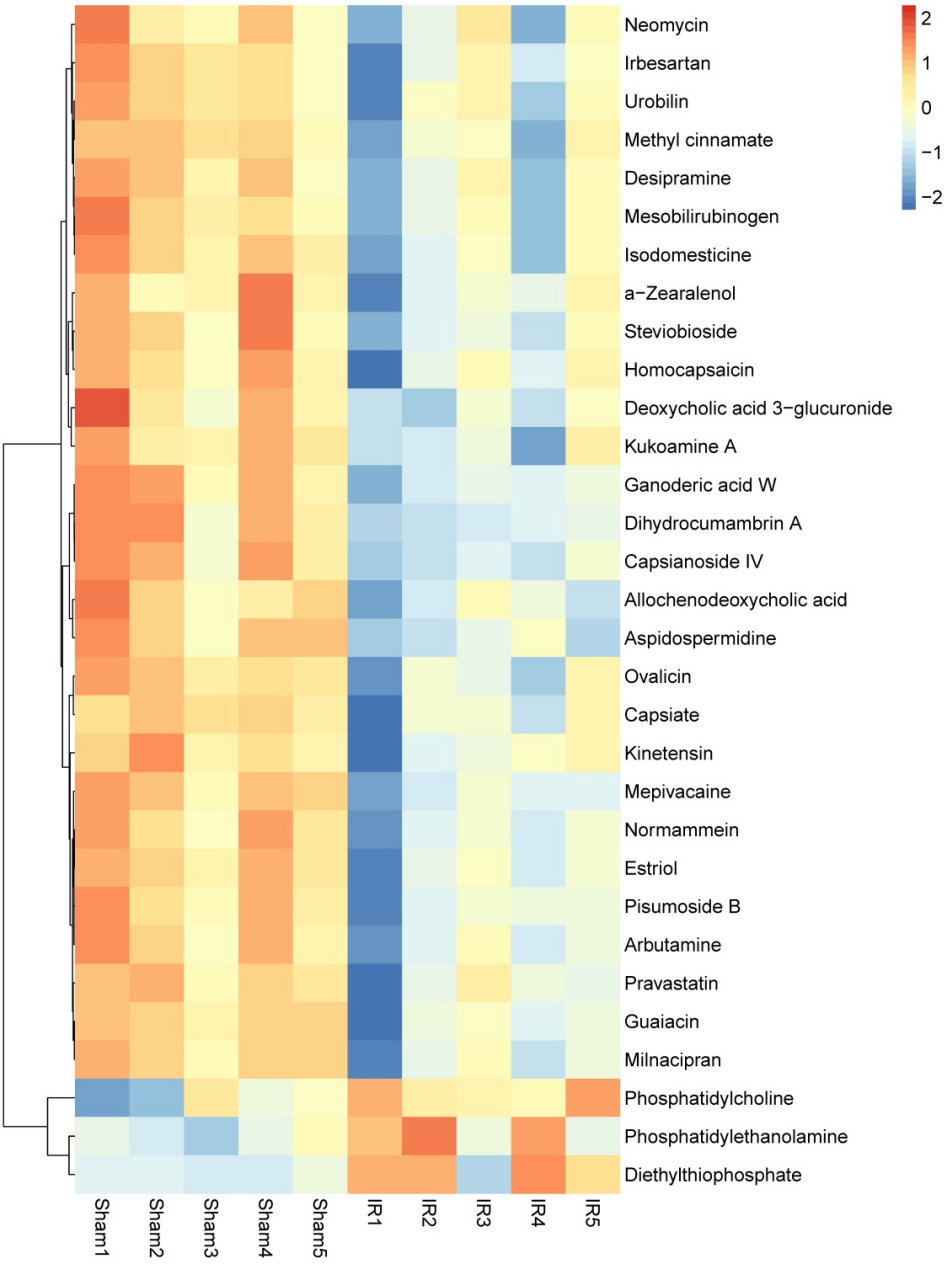
The changes of metabolites in intestinal I/R.

In recent years, the potential role of intestinal microbiota and its metabolites in the development of various human diseases has attracted considerable attention. We summarize the changes in the composition of intestinal microbiota and its metabolites in intestinal I/R injury (Table 2 and Figure 1). Wang *et al.* found that intestinal I/R affect the bacterial structure of the rat's colon. The colonic microbiota began to change as early as 1 hour after reperfusion, and reached the most obvious bacteria structure change at 6 hours after reperfusion. Among them, the abundance of *Escherichia coli* significantly increased at 1 hour and 3 hours of reperfusion; the content of *Lactobacillus* increased significantly after 6 hours of reperfusion; the abundance of *Oral Prevotella* was significantly increased from 1 hour to 12 hours after reperfusion 55. The research team also reported the imbalance of the ileal bacteria in rats caused by intestinal I/R. The ileal bacteria changed at the beginning of reperfusion, and the most obvious difference appeared at 12 hours of reperfusion, and then gradually returned to normal. Specific ecological disorders are characterized by the proliferation of *Escherichia coli* and the decrease of *Lachnospiraceae* and *Lactobacillus*, and changes in the ileal bacteria follow epithelial changes 56. Deng *et al.* used 16S rRNA combined with metabonomics to clarify the changes in intestinal bacteria and metabolites induced by mouse intestinal I/R. The study has shown that the composition of the colonic bacteria is significantly disordered after intestinal I/R in mice. The relative abundance of *Firmicutes* and *Bacteroidetes* increases significantly, while the relative abundance of *Verrucomicrobia* was reduced. At the species level, the relative abundance of *Bacteroides vulgatus* and *Parabacteroides distasonis* increased after I/R. Metabonomics results showed that the biosynthesis of secondary metabolites and polysaccharides after I/R and the genomic abundance of metabolic pathways are significantly impaired, and the content of metabolites of microbiota such as capsiate and pravastatin occurs change (Figure 2) 57.

Intestinal I/R injury will not only involve local intestinal tissue injury, but also the secondary bacterial migration, inflammatory factors and endotoxin release after the intestinal barrier disorder will cause damage to the extraintestinal organs. Therefore, accurately revealing the changes of intestinal microbiota and its metabolites in intestinal I/R injury will not only help clarify the mechanism of intestinal and extraintestinal organ injury induced by intestinal I/R, but also seek the biomarker of intestinal microbiota and metabolites for predicting, diagnosing and preventing intestinal I/R-induced intestinal injury and extraintestinal organ injury; and help to find effective drugs for the treatment of intestinal and extraintestinal organ injury induced by intestinal I/R.

## The overall effect of microbiota on intestinal I/R

While the intestinal I/R injury changes the composition of intestinal microbiota and its metabolites, on the contrary, studies have found that overall changes of microbiota, usually by antibiotics and fecal microbiota transplantation (FMT) treatment, also affect the outcome of intestinal I/R injury (Table 3).

### Antibiotics

Intestinal dysbacteriosis and changes in metabolites are causal to each other in intestinal and extraintestinal organ injury induced by intestinal I/R. Because the role of specific bacteria strains and their metabolites in intestinal I/R-induced intestinal and extraintestinal organ injury has not been fully elucidated, and the intervention of specific bacteria strains still lacks effective measures; in addition, intestinal bacterial translocation may increase the level of inflammatory factors and endotoxins in the blood. Therefore, many factors suggest that the use of antibiotics may be an effective measure to reduce the intestinal and extraintestinal organ injury induced by intestinal I/R. Studies have found that using antibiotics to consume intestinal commensal bacteria reduces the expression of B cells, immunoglobulins (Igs) and Toll-like receptors (TLRs) in the intestine, inhibit complement activation, and reduce intestinal inflammation and injury after intestinal I/R 58. During intestinal I/R, symbiotic bacteria activate and attract inflammatory cells such as neutrophils, macrophages and lymphocytes, which cause intestinal inflammation and aggravate I/R-induced intestinal injury. Ascher *et al.* revealed that antibiotic treatment reduces lymphatic tissue and the deposition of immunoglobulin and complement, reduces intestinal inflammation, and improves intestinal integrity 59. Rapamycin treatment improved the survival rate of mice, and reduced of lung bacteria and increase in phagocytic activity after intestinal I/R 60. However, some studies have also reported that antibiotic treatment aggravated intestinal I/R injury. Ascher *et al.* found that antibiotic pretreatment reduces leukocyte adhesion, but increases NETing neutrophils, which is because neutrophil TLR4/TRIF signal-mediated intestinal I/R injury is not conducive to the recovery of NETosis 59. Zhang *et al.* uncovered that the commensal bacteria enhance the proliferation and migration of intestinal epithelial cells, and the absence of commensal bacteria eliminated the inflammatory response in the early stage, but failed to improve the overall survival after intestinal I/R 61. Therefore, the above-mentioned use of antibiotics in intestinal I/R injury is currently controversial, which may be related to the complexity of the intestinal microbiota, the angle of observation and the difference in time.

### Fecal microbiota transplantation

Adjusting or improving disease-related microbiota imbalance or disorder through FMT has always been an effective direction and strategy for the treatment of diseases. Franziska Bayer *et al.* reported that transplanting altered Schaedler flora from C3H/HeNTac mice reduces the adhesion of leukocytes to the endothelium of mesenteric venules before and after intestinal I/R, and reduces the vascular inflammation induced by intestinal I/R 62. Therefore, FMT is a potentially effective treatment for intestinal I/R injury.

## The role of intestinal microbiota and its metabolites in intestinal and extraintestinal organ injury induced by intestinal I/R

In recent years, more and more studies have found that intestinal microbiota and its metabolites play an important role in the treatment of intestinal and extraintestinal organ injury induced by intestinal I/R (Table 4).

### Intestinal bacteria

With the use of probiotics, more and more intestinal bacteria strains were discovered to have the potential to treat intestinal and extraintestinal organ injury induced by intestinal I/R. *Bifidobacterium bifidum PRL2010* was found to reduce intestinal I/R injury, inhibit neutrophil infiltration, especially at the lung level, moderately reduce oxidative stress, significantly reduce bacterial translocation, and down-regulate the transcription level of inflammatory factors in the liver and kidney 63. Wang *et al.* found that *Bifidobacteria* inhibits I/R-induced the apoptosis of intestinal epithelial cells, the destruction of tight junctions and bacterial translocation, and reduces the release of pro-inflammatory cytokines and endotoxins, and increases the concentration of SCFA, restores the microbial community structure and the integrity of the mucosa 64. *Lactobacillus plantarum* reduces intestinal I/R injury and inflammation, prevents intestinal epithelial cell apoptosis, and effectively prevents bacterial translocation 65. Long-term feeding of probiotic VSL#3 improves intestinal I/R injury by reducing leukocyte recruitment and pro-inflammatory cytokines 66. *Lactobacillus murinus* relieves intestinal I/R injury by activating TLR2 signal and promotes the release of Interleukin-10 (IL-10) from M2 macrophages, which can significantly prevent intestinal I/R injury. In addition, correlation analysis showed that clinically, the abundance of *Lactobacillus murinus* in the feces of patients undergoing cardiopulmonary bypass surgery is closely related to the degree of postoperative intestinal I/R injury 67. The combined use of *L. plantarum DSM 9843* and rose hip reduces cecal I/R injury, the oxidative stress level of cecal tissue and the abundance of e*nterobacter* 68. Serum transfer from young wild mice with bacteria reverses the sterile inflammatory injury caused by the intestinal I/R of sterile mice, which may be related to the restoration of IgG deposition, leukocyte influx, NF-κB activation and pro-inflammatory gene expression in inflamed tissues, while down-regulating the production of Annexin-1 and IL-10 69.

Not only that, some strains have also been discovered to promote intestinal I/R injury. The presence of *Pseudomonas aeruginosa* in the distal intestine may enhance the lethal effect of intestinal I/R on mice, partly due to the *in vivo* virulence activation of the epithelial barrier destroying protein PA 1 lectin 70. Single colonization of *Escherichia coli strain JP313* enhanced the degree of leukocyte adhesion to the mesenteric venules damaged by I/R 59. What's more, certain strains, like *E. coli*, affect the level of injury to extra-intestinal organs induced by intestinal I/R. Wen et al. found that the gram-negative gut pathobiont *E. coli* translocated into livers, as a result of barrier disruption, and was present in the hepatic sinusoid and close to the endothelium, suggesting bacterial translocation-induced hepatic damage after intestinal I/R injury 71. Xu *et al.* found that brain ischemia rapidly induced intestinal ischemia and *Enterobacteriaceae* expansion, exacerbating brain infarction in turn by enhancing systemic inflammation 72.

### Metabolites of intestinal microbiota

In addition to specific intestinal bacteria strains, intestinal microbiota metabolites have also been confirmed to play an important role in intestinal and extraintestinal organ injury induced by intestinal I/R.

#### Short-chain fatty acids

SCFA are the main bacterial metabolites produced by specific colonic anaerobes after fermenting dietary fiber and resistant starch, mainly including acetate, propionate and butyrate 73. SCFA are signaling molecules that mediate the interaction between diet, intestinal microbiota and the host, and play an important role in the body's immunity, metabolism, and endocrine 74. SCFA regulates intestinal and host metabolism by activating G protein-coupled cell surface receptors G protein-coupled receptor 41 (GPR41) and GPR43 75. These two SCFA receptors are not only expressed in the intestine, but also in human fat, muscle and liver tissues, indicating that SCFA can directly regulate substrate and energy metabolism in peripheral tissues. SCFA protects the distal small intestinal mucosa during intestinal I/R and reduces the infiltration of neutrophils into the intestinal lamina propria 76. Schofield *et al.* reported that acetate reduces intestinal I/R injury through GPR43 77. Qiao *et al.* revealed that butyrate administration reduces intestinal I/R injury, which is related to protecting the intestine tight junction barrier and inhibiting the infiltration of inflammatory cells in the intestinal mucosa 78. Therefore, SCFA plays an important role in maintaining intestinal barrier homeostasis and reducing mucosal inflammation during intestinal I/R.

#### Secondary bile acids

Bile acids play an important role in the body's lipid metabolism 79, 80. After a meal, primary bile acids enter the intestinal tract along with bile. In the upper part of the intestine, bile acids can regulate the digestion and absorption of lipids; primary bile acids can be converted to secondary bile acids, including deoxycholic acid and lithocholic acid, by removing hydroxyl groups under the action of intestinal bacteria in the lower part of the intestine (ileum and proximal colon), some of which will also be reabsorbed into liver. Bile acids have a great influence on the structural composition of the intestinal flora. Bile acids can combine with the phospholipids on the bacterial cell membrane to play a destructive effect, and resist bacterial adhesion and neutralize endotoxins. The high concentration of bound bile acid has a direct antibacterial effect 81. At the same time, the intestinal flora also plays a key role in the bile acid cycle. The bacteria in the intestine dissociate the conjugates of taurine, glycine, sulfate, etc. in the primary bile acid through bile salt hydrolase, change its chemical properties, and regulate the body's lipid metabolism. Bile acid receptors include nuclear receptors and membrane receptors, the former includes farnesoid X receptor (FXR), pregnane X receptor (PXR), and vitamin D receptor (VDR), while the latter refers to G protein-coupled bile acid receptor 1 (GPBAR1), also known as Takeda G-protein-coupled receptor 5 (TGR5) 82, 83. After intestinal I/R, the expression levels of FXR and PXR in the intestinal tissue and liver are significantly reduced, and IL-6 is one of the main reasons for the decreased expression of these receptors 84. Pretreatment with FXR agonist OCA improves the survival rate of caries animal intestinal I/R model, protects the intestinal barrier function and inhibits inflammation 85. The FXR agonist CSE reduces intestinal I/R injury by inhibiting the inflammatory response and the NF-κb pathway 86.

#### Tryptophan metabolites

In many aspects such as the intestinal barrier, intestinal immunity and endocrine function, and intestinal motility, intestinal tryptophan and its metabolites interact closely with the intestinal microbiota 87, 88. There are three metabolic pathways of tryptophan in the human intestine: first, in intestinal epithelial cells and immune cells, about 90% of tryptophan is metabolized to kynurenine by indoleamine 2,3-dioxygenase 1 (IDO1); secondly, in the intestinal lumen, the intestinal flora directly metabolizes about 4--6% tryptophan; thirdly, about 3% tryptophan in enterochromaffin cells is metabolized to serotonin via tryptophan hydroxylase 1, the serotonin (5-HT) pathway, which produces more than 90% of the body's 5-HT 89, 90. Intestinal tryptophan metabolites also affect various intestinal functions such as barrier, peristalsis, digestion and absorption, secretion, immunity, etc. There have been some studies reporting the role of 5-HT in intestinal I/R injury. Potentiation of 5-HT signaling is associated with mucosal protection from intestinal I/R injury without alterations in villus cell distribution, possibly via increased rates of enterocyte renewal 91. Knock-out of serotonin re-uptake transporters (SERT) or use of selective serotonin re-uptake inhibitors (SSRIs) significantly decreased mucosal injury and inflammation after intestinal I/R 91. However, sumatriptan may regulate inflammation by activating 5-HT1B/1D receptors, thereby inhibiting I/R-induced intestinal injury 92. Intestinal I/R induces continuous disturbance of liver microcirculation, leading to liver dysfunction. 5-HT may be one of the mediators of liver dysfunction after intestinal I/R 93. Therefore, the role of 5-HT in intestinal I/R injury is still controversial.

Furthermore, Aryl hydrocarbon receptors (AHR), a ligand-dependent transcription factor and widely expressed in immune, epithelial, endothelial and stromal cells in barrier tissues, mediate the regulation of intestinal immunity and barrier homeostasis by tryptophan metabolites. Jing* et al.* found that regulating AHR expression may reduce liver injury induced by intestinal I/R 94. AHR activation improves epithelial barrier dysfunction following intestinal I/R 95, 96. Furthermore, AHR has been proved to be key receptors that promote host defense and enhance disease tolerance of endotoxemia 97. Therefore, AHR is a potentially effective target for the treatment of intestinal I/R injury.

#### Other

In addition to several typical types of intestinal microbiota metabolites, other proven intestinal microbiota metabolites have also been observed to have a significant effect on intestinal I/R injury. We have uncovered that gut microbiota metabolite capsiate (CAT) enhances glutathione peroxidase 4 (GPX4) expression and inhibits ferroptosis by activating transient receptor potential cation channel subfamily V member 1 (TRPV1) in intestinal I/R injury, providing a potential avenue for the management of intestinal I/R injury. Pravastatin (PA), a metabolite of intestinal microbiota, promotes the release of IL-13 from type II innate lymphoid cells (ILC2s) through IL-33/ST2 signals. And IL-13 promotes the self-renewal of intestinal stem cells by activating Notch1 and Wnt signals, and ultimately reduces intestinal I/R injury. In addition, the PA content in the feces of patients before CPB surgery promotes patients to resist postoperative intestinal I/R injury 98. Tian *et al.* reported that polyunsaturated fatty acids (PUFAs), especially n-3 PUFAs, improve the function of the intestinal barrier by regulating the innate immunity after intestinal I/R 99. Sileri *et al.* confirmed that a single injection of exogenous Melatonin significantly reduces intestinal I/R injury and prevents bacterial translocation 100. Furthermore, fasting for two days without I/R injury will not cause mucosal changes and bacterial translocation, but in the case of intestinal I/R injury, fasting for two days will increase the susceptibility of bacterial translocation to the rat's systemic organs 101. Neutrophils induced by hypoxic pretreatment prevent I/R-induced bacterial translocation through antibacterial activity and promotion of epithelial barrier integrity 102. Glutamine and glucan reduce bacterial translocation and cytokine release levels, thereby reducing intestinal injury 103. Beta-(1-3)-D-glucan regulates the production of pro-inflammatory and anti-inflammatory cytokines in the intestinal I/R and reduces bacterial translocation 104. Anti-CINC antibody treatment reduces the infiltration of small intestinal neutrophils and the degree of mucosal damage, inhibits inflammation, reduces bacterial translocation, and protects the small intestine from I/R injury 105. Therefore, maintaining intestinal barrier homeostasis and reducing intestinal barrier permeability are effective measures to reduce bacterial translocation.

## Conclusion

As emphasized in this review, intestinal I/R can cause imbalance of the intestinal microflora; the intestinal microbiome is also involved in the development of intestinal I/R and affects the level of injury to extraintestinal organs. Strengthening the intestinal barrier function is a good way to reduce the translocation of bacteria and bacterial metabolites and reduce the damage of extraintestinal organs induced by intestinal I/R. The intestinal microbiota includes bacteria, fungi, viruses, and archaea. Although more than 90% of the intestinal flora are bacteria, the role of viruses, fungi and archaea in intestinal I/R damage cannot be limited. Since the studies on the role of metabolites of intestinal microbiota cited in the review were mainly animal studies, more clinical studies are needed to confirm these findings. The detailed role of these microorganisms in the damage of intestinal I/R and its extraintestinal organs needs further study. It remains to be seen whether the composition and changes of the intestinal microbiome can be used as biomarkers of intestinal I/R and its extraintestinal organ damage. This review hopes that a more detailed understanding of the changes, effects and mechanisms of the intestinal microbiota in intestinal I/R injury will help to develop effective methods to reduce the incidence and mortality of intestinal I/R injury.

## Figures and Tables

**Table 1:** The risk factors of intestinal I/R injury during perioperative period

Factor	
Patient factors	Advanced age
	ASA grade ≥ III
	Preoperative gastrointestinal disease
	Other diseases that lead to impaired gastrointestinal function (such as severe infection, acute severe pancreatitis, trauma, shock, anemia, myocardial infarction, aortic dissection, mesenteric artery embolism, etc.)
Anesthetic factors	Hypotension and intestinal hypoperfusion caused by anesthetics
	Vasoconstrictor drugs that cause small blood vessels to constrict in the gastrointestinal mucosa
	Sympathetic nervous system excitement
Surgical factors	Abdominal aortic aneurysm surgery
	Cardiopulmonary bypass
	Abdominal surgery and laparoscopic surgery
	Other intestinal operations that affect intestinal blood perfusion

**Table 2:** Microbiota changes in intestinal I/R injury

Species	Intestial site	Microbiota changes	Main results	Ref.
Rat	Colon	*Diversity* ↑ *Escherichia coli* ↑ *Lactobacillus* ↑ *Prevotella oralis* ↑	Dysbiosis and tendency of recovery of colonic microbiota after damage and repair of the epithelium.	55
Rat	Ileum	*Diversity* ↑ *Escherichia coli* ↑ *Lactobacilli* ↑ *Prevotella* ↑ *Lactobacillus* ↓ *Lachnospiraceae* ↓ *Prevotella* ↓	Earlier damage and repair in the ileal epithelium compared with later dysbiosis and recovery of ileal microbiome.	56
Mice	Cecum	*Diversity* ↑ *Bacteroides vulgatus* ↑ *Parabacteroides distasonis* ↑ *Verrucomicrobia* ↓ *Capsiate (One microbiota metabolite)* ↓	Capsiate enhances Gpx4 expression and inhibits ferroptosis by activating TRPV1 in intestinal I/R injury	57

**Table 3:** The overall effect of microbiota on intestinal I/R

Intestinal microbiota treatment		Species	Main effects	References
Antibiotic treatment & germ-free mice	Antibiotic treatment	Mice	Depletion of gut commensal bacteria attenuated intestinal inflammation and injury following I/R.	58
	Antibiotic treatment & germ-free mice	Mice	Gut microbiota suppressed NETing neutrophil hyperreactivity in mesenteric I/R injury, while ensuring immunovigilance by enhancing neutrophil recruitment.	59
	Rapamycin antibiotic treatment	Mice	Rapamycin treatment improved mortality following intestinal I/R via the inhibition of remote lung inflammation in mice.	60
	Ampicillin antibiotic treatment	Mice	Depletion of commensal bacteria by ampicillin resulted in inhibition of injury, neutrophil infiltration, and TNF-α expression.	106
	Antibiotic treatment	Mice	Commensal bacteria deletion improved mice survival in the early phase, but failed to improve the overall survival at 96 h after intestinal I/R.	61
	Germ-free mice	Mice	The lack of intestinal microbiota is accompanied by a state of active IL-10-mediated inflammatory hyporesponsiveness.	107
FMT	Schaedler flora (ASF)	Mice	ASF-colonized mice showed reduced leukocyte adherence in I/R injury.	62

**Table 4:** The role of intestinal microbiota and its metabolites in intestinal and extraintestinal organ injury induced by intestinal I/R

Intestinal microbiota treatment		Species	Main effects	References
Bacterial strain	*B. bifidum PRL2010*	Mice	*B. bifidum PRL2010* reduced bacterial translocation, transcription levels of TNFalpha and IL-10 both in liver and kidneys and neutrophil recruitment in the lungs.	63
	*Bifidobacteria*	Mice	Pretreatment of animals with *bifidobacteria* prevented I/R-induced bacterial translocation, reduced pro-inflammatory cytokine release, the levels of endotoxin, intestinal epithelial cell apoptosis, disruption of tight junction and increased the concentration of SCFA, resulting in recovered microbiota and mucosal integrity.	64
	*Lactobacillus plantarum L2*	Rats	*Lactobacillus plantarum* prevented I/R induced bacterial translocation, reduced pro-inflammatory cytokine release, and intestinal epithelial cell apoptosis.	65
	Probiotic VSL#3	Mice	VSL#3 reduced local tissue injury from I/R by down-regulating pro-inflammatory mediators and immune cell recruitment.	66
	*Lactobacillus plantarum DSM 9843* and rose hip	Mice	Decreased MDA levels in cecum tissue and Enterobacteriaceae counts in cecun stool.	68
	Microbiota induced natural Abs	Mice	Microbiota induced natural Abs restored IgG deposition, leukocyte influx, NF-κB activation, and proinflammatory gene expression and concomitantly downregulated annexin-1 and IL-10 production.	69
	*Pseudomonas aeruginosa*	Mice	*P. aeruginosa* potentiates the lethal effect of IR in mice in part due to *in vivo* virulence activation of its epithelial barrier disrupting protein PA-IL.	70
	*Escherichia coli*	Mice	*Escherichia coli* augmented leukocyte adhesion to the ischemia-reperfusion injured endothelium of mesenteric venules.	59
	*Bifidobacteria*	Mice	*Bifidobacteria* prevented I/R-induced bacterial translocation, reduced pro-inflammatory cytokine release, the levels of endotoxin, intestinal epithelial cell apoptosis, disruption of TJ and increased the concentration of SCFA, resulting in recovered microbiota and mucosal integrity.	64
Gut metabolites	SCFA (butyrate, propionate and acetate)	Rats	SCFA protect the distal small bowel mucosa and diminishes infiltration of neutrophils to the gut lamina propria in intestinal I/R.	108
	SCFA (acetate)	Mice	Acetate treatment to WT mice prior to ischemia protected the intestine from I/R-induced damage protective, which were GPR43-independent.	77
	SCFA (butyrate)	Rats	Butyrate attenuated the inflammatory factor levels and leukocyte infiltration, maintained the intestinal barrier structures, increased the expression of tight junction proteins, and decreased endotoxin translocation.	78
	FXR agonist (obeticholic acid)	Rats	Obeticholic acid improved survival in a rodent model of intestinal I/R injury, preserved the gut barrier function and suppressed inflammation.	85
	FXR agonist (INT-747)	Mice	FXR activation enhanced intestinal epithelial cell tolerance to I/R by suppressing the inflammatory response and NF-κB pathway via cystathionine-γ-lyase mediation.	86
	Capsiate	Mice	Capsiate enhanced Gpx4 expression and inhibited ferroptosis by activating TRPV1 in intestinal I/R injury.	57
	Pravastatin	Mice	Pravastatin attenuated intestinal I/R injury by promoting the release of IL-13 from type II innate lymphoid cells *via* IL-33/ST2 signaling.	98
Drugs	N-3 polyunsaturated fatty acids (PUFAs)	Rats	N-3 PUFAs protect the intestinal barrier by modifying intracellular I-FABP, activating the PPARg pathway, and then upregulating tight junction protein expression.	109
Others	Melatonin	Rats	Melatonin significantly reduced the intestinal IR injury and prevented bacterial translocation.	100
	Two-day fasting	Rats	Two-day fasting increased the susceptibility of bacterial translocation to systemic organs in I/R injury.	101
	Hypoxic preconditioning (HPC)	Rats	Neutrophil priming by HPC protected against I/R-induced BT via direct antimicrobial activity by oxidative respiratory bursts and through promotion of epithelial barrier integrity for luminal confinement of enteric bacteria.	102
	Glucan and glutamine	Rats	Glucan and glutamine reduced bacterial translocation, intestinal damage, and cytokine levels after I/R.	103
	Beta-(1-3)-D-glucan	Rats	Beta-(1-3)-D-glucan modulated the production of pro-inflammatory and anti-inflammatory cytokines during bowel ischemia/reperfusion, and attenuated translocation of labelled bacteria.	104

## Abbreviations

I/R: ischemia/reperfusion
ASA: American Society of Anaesthesiologists
CPB: cardiopulmonary bypass
ITS: Internally Transcribed Spacer
Igs: immunoglobulins
TLRs: Toll-like receptors
FMT: Fecal microbiota transplantation
IL-10: Interleukin-10
SCFA: Short-chain fatty acids
GPR41: G protein-coupled receptor 41
FXR: farnesoid X receptor
PXR: pregnane X receptor
VDR: vitamin D receptor
GPBAR1: G protein-coupled bile acid receptor 1
TGR5: Takeda G-protein-coupled receptor 5
IDO1: indoleamine 2,3-dioxygenase 1
SERT: serotonin re-uptake transporters
SSRIs: selective serotonin re-uptake inhibitors
AHR: Aryl hydrocarbon receptors
CAT: capsiate
GPX4: glutathione peroxidase 4
TRPV1: transient receptor potential cation channel subfamily V member 1
PA: Pravastatin
ILC2s: type II innate lymphoid cells
PUFAs: polyunsaturated fatty acids
